# A seven week observational analysis of clinical activities in a North Italian orthopaedic hospital during the second wave of SARS-CoV-2 pandemic: far from usual volumes, but different from the first wave

**DOI:** 10.1007/s00264-021-05064-8

**Published:** 2021-05-13

**Authors:** Luigi Zagra, Martina Faraldi, Mauro Andreata, Immacolata Ottaiano, Giuseppe Basile, Giovanni Lombardi

**Affiliations:** 1 Hip Department, IRCCS Istituto Ortopedico Galeazzi, Milan, Italy; 2 Laboratory of Experimental Biochemistry and Molecular Biology, IRCCS Istituto Ortopedico Galeazzi, Milan, Italy; 3 IRCCS Istituto Ortopedico Galeazzi, Milan, Italy; 4 ER Department, IRCCS Istituto Ortopedico Galeazzi, Milan, Italy; 5 Department of Athletics, Strength and Conditioning, Poznań University of Physical Education, Poznań, Poland

**Keywords:** COVID-19, Coronavirus, Orthopaedic surgery, Orthopaedic trauma, Healthcare systems

## Abstract

**Purpose:**

We previously described the radical changes occurred in an orthopaedic hospital in Milan (Italy) during the first SARS-CoV-2 pandemic outbreak. Currently, during the second wave, the situation is still far from normality. Here we describe the changes that took place, and are still ongoing, in the clinical practice.

**Methods:**

Number and type of admissions, outpatients activity, ER and urgent procedures in SARS-CoV-2 negative and positive patients have been analyzed over seven weeks (October 26th–December 13th, 2020) and compared with the correspondent period in 2019 and the same timeframe during the first wave (February 24th–April 10th).

**Results:**

2019 vs. 2020: Overall admissions decreased by 39.8%; however, while admissions for elective surgery dropped by 42.0%, urgent surgeries increased by 117.0%. Rehabilitation admissions declined by 85.2%. White and green priority ER consultations declined by 41.6% and 52.0%, respectively; yellow and red increased by 766.7% and 400.0%, respectively.

Second vs. first wave: Overall admissions increased by 58.6% with a smoother decrement in weekly admissions than during the first wave.

Disparity of acute admissions vs. rehabilitation expanded: Acute cases increased by 63.6% while rehabilitation cases decreased by 8.7%. Admissions to triage procedures increased by 72.3%.

**Conclusions:**

Activity levels are far from normality during the second COVID-19 wave. Elective surgery and outpatients-related activities are still strongly limited compared to 2019 while the number of urgent cases treated increased consistently. SARS-CoV-2 positive emergencies are slightly higher than during the first wave. These important changes are expected to impact on health service and hospital budget for long.

## Introduction

Until today, the SARS-CoV-2 pandemic has been characterized, worldwide, by two main waves of infection outbreak intervalled by a period of slowdown, corresponding to the late spring–summer of the boreal hemisphere, linked to the season and the effects of the containment measures implemented by local authorities. Italy was the first European country to be overwhelmed by the first pandemic wave, at the beginning of March 2020. Northern Italy, and in particular the Lombardia region, has been the epicentre. Severe containment measures have allowed a substantial reduction in viral transmission from May until October 2020. However, a second wave has subsequently invested the whole country determining high rates of infection and, consequently, mortality [1]. Waiting for a relevant reduction in the transmission of the disease thanks to the mass vaccination campaigns, we are still witnessing the stress on health structures for a period to come still uncertain [2].

IRCCS Istituto Ortopedico Galeazzi is a high-volume clinical and research hospital located in Milan, the main city of Lombardia, which is the largest Italian region (more than 10 million inhabitants recorded at the beginning of 2020). We had previously analysed some key indicators (i.e. orthopaedic clinical practice [3], arthroplasty practice [4], operating room efficiency [5]) of the hospital activity during the clue seven week timeframe of the first epidemic compared to the same period of the previous year (i.e. 2019). After the first pandemic wave, a slow progressive return towards usual activities occurred, according to international guidelines that acknowledged the reinstating elective orthopaedic surgery [6] with a huge attention on well-being and safety of patients [7] according to stringent protocols that contemplate the SARS-CoV-2 screening for all the patients [8] and the prioritisation of the patients according to the intervention needed [9]. The advent of a second wave of infections has brought back to light criticalities similar to those emerged during the first part of the year although with some peculiarities. According to regional authorities, Galeazzi Institute became, again, as during the first wave, one of the two reference centres for minor trauma while general hospitals dramatically reduced their surgical activity and nearly stopped elective surgery in order to reassign the personnel to COVID areas.

This study aims to describe the persistent as well as the new changes that impacted again on diagnosis and treatment of orthopaedic patients during the second epidemic wave in a specialized orthopaedic centre in Milan. A period of seven weeks, from October 26th to December 13th, has been chosen to be compared with the same period of 2019 and with the corresponding timeframe during the first wave.

## Materials and methods

On October 23rd, the regional health authorities of Lombardia detected a worsening of the SARS-CoV-2 outbreak. The organisational health structure was increased from level 1 to level 3 to face the resurgence of COVID-19 epidemic. Hospitals had to suspend all the activities of planned admission to provide enough rooms for COVID-19-related activities. Urgent cases had to be redistributed within specific networks (acute myocardial infarction [AMI], stroke, trauma, vascular surgery), similarly to what happened during the first wave, since the second week of March.

Galeazzi Orthopaedic Institute had to be ready to accept minor trauma cases from neighbouring hospitals as well as non-postponable orthopaedic patients coming from general hospitals that were facing with the network reorganization. The emergency department sustained an unprecedented increment in admissions, mainly femoral fractures, referred from nearby hospitals. The reorganization continued with dedicated pathways for SARS-CoV-2-positive patients admitted from the emergency room (ER), through a filter area before being assigned to either COVID-19 or non-COVID-19 departments and surgical rooms.

During the second epidemic wave, the elective surgical activity was heavily reduced but not completely abolished. Nevertheless, the reduced surgical slots forced the surgeons to give priority to the most complex and undeferrable cases and, specifically, periprosthetic joint infections, prosthetic loosening with high-grade osteolysis, rapidly progressive osteoarthritis, osteonecrosis.

The outpatients department, whose activity volumes, during the period between the pandemic waves, never reached the pre-COVID-19 level, had no cancelled cases but experienced a mild and spontaneous decrement in the total number of weekly visits.

The rehabilitation department experienced a steep reduction in the number of beds available due to both the reallocation of medical and nurse staff and to the strict safety protocols adopted to limit the risk of virus transmission.

Internal administrative flows integrated and double checked by the health management data of the hospital represented the data sources of the present study, updating the database analysed in our previous study [3]. Approval was obtained by the institutional review board in using anonymous data flows.

### Statistical analysis

Data of 2019 and 2020 and data of the first wave (from February 24th to April 10th, 2020) and the second wave (from October 26th to December 13th, 2020) were analysed trough a *χ*^2^ test. Comparison between data over the seven week timeframe in 2020 and the corresponding period in 2019, and comparison between data over the seven weeks during both the first wave and the second wave were performed trough the Mann–Whitney *U* test. Statistical analysis were performed on GraphPad Prism v6.0 (GraphPad Software, Inc., La Jolla, CA, USA).

## Results

### Comparison between 2020 and 2019

**Fig. 1:**
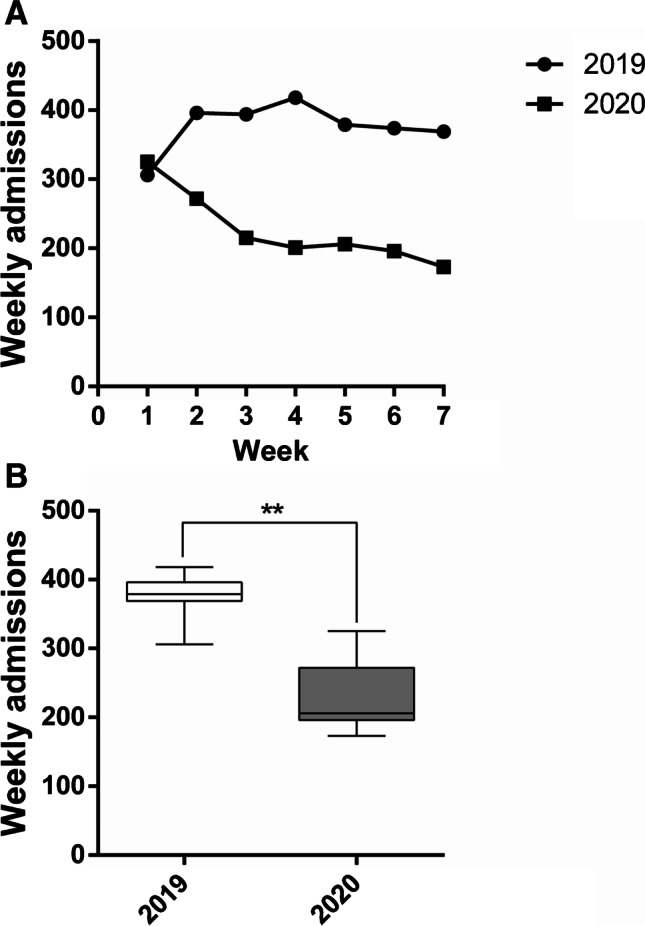
Admissions from October 26th to December 13th, 2020, compared to the corresponding period in 2019. **A** Weekly admission trends during the considered 7-week timeframe in 2020 and 2019. **B** Mean number of weekly admissions in 2020 compared to 2019. Data are reported as minimum to maximum, and were compared trough the Mann–Whitney *U* test. Comparison is significant when *p* < 0.05

**Fig. 2:**
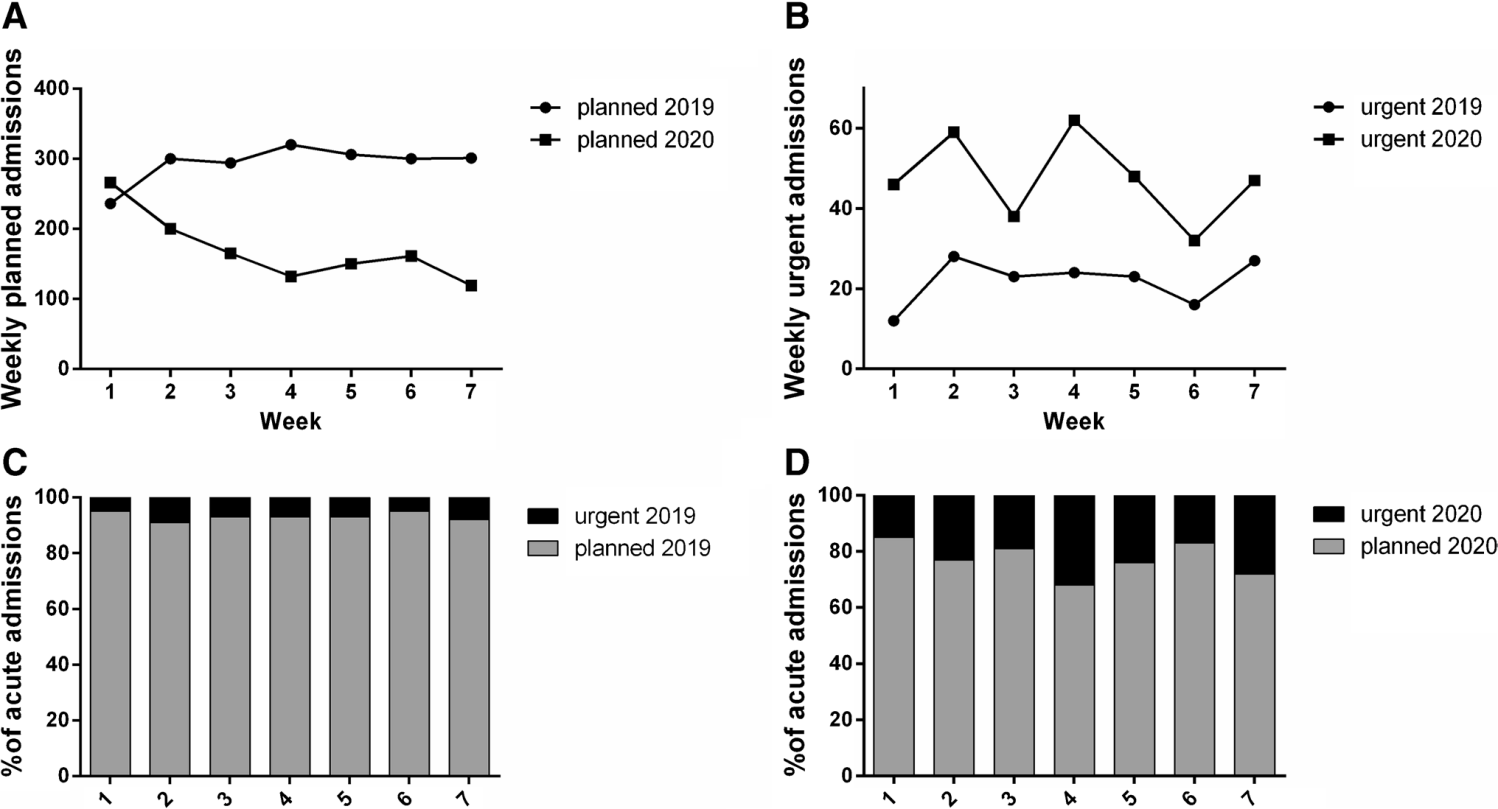
Planned and urgent surgery from October 26th to December 13th, 2020, compared to the corresponding period in 2019. **A** Weekly trends of planned surgery in 2020 and 2019. **B** Weekly trends of urgent surgery in 2020 and 2019. **C** Percentage of weekly planned and urgent surgery in 2019. **D** Percentage of weekly planned and urgent surgery in 2020

A seven week period during the second SARS-CoV-2 pandemic wave (October 26th to December 13th, 2020) was compared with the corresponding period in 2019. The total number of admissions in 2020 (1588) was decreased by 39.8% compared to those recorded in 2019 (2636, *p* < 0.0001; Table 1). Total admissions in 2020 declined throughout the seven week period (Table 2; Fig. 1A), and the mean weekly numbers of admissions were significantly different (226.86 ± 52.86 in 2020 vs. 376.57 ± 35.23 in 2019, *p* = 0.001) (Fig. 1B). The difference was confirmed when grouping for type of admission, either surgical or rehabilitative, that decreased by 31.0% and 85.2%, respectively (Table 1). However, among the surgical admissions, while those for elective surgery dropped by 42.0% (from 2058, in 2019, to 1193, in 2020), those for urgent surgery (mainly from the emergency department) increased by 117.0% (from 153, in 2019, to 332, in 2020) (Table 3; Figs. 2A and B). Moreover, while in 2019 the surgical activity of the hospital remained constant throughout the 7-week period taken into consideration, in 2020, there was a certain degree of variability, among both elective and urgent surgery (Figs. 2C and D).

**Table 1:** Overall admissions during a 7-week period in 2020 vs.2019 and in second wave vs. first wave

	Type of admission	No. per type of admission	No. of admissions	% of admissions	*χ*^2^ test df (1)	*p* value
2020 vs.2019						
2019	Surgery	2211	2636	83.9%	143.1	< 0.0001
	Rehabilitation	425		16.1%		
2020	Surgery	1525	1588	96.0%		
	Rehabilitation	63		4.0%		
Second wave vs. first wave						
First wave	Surgery	932	1001	93.1%	10.9	0.001
	Rehabilitation	69		6.9%		
Second wave	Surgery	1525	1588	96.0%		
	Rehabilitation	63		4.0%		

Comparison in the number of overall admissions, considering both surgery and rehabilitation admissions, between the period October 26th-December 13th, 2020, and the same timeframe in 2019 (2020 vs. 2019) and between October 26th and December 13th, 2020, and February 24th and April 10th, 2020 (second wave vs. first wave). Data are reported as absolute and percentage values and compared through the Pearson’s Chi-squared test. Respective Chi-squared tests and *p* values are shown. *p* < 0.05 is considered statistically significant

**Table 2:** Overall weekly admissions in 2020 vs.2019 and in second wave vs. first wave

	Week 1	Week 2	Week 3	Week 4	Week 5	Week 6	Week 7	*χ*^2^ test df (6)	*p* value
2020 vs. 2019									
2019	306	396	394	418	379	374	369	74.5	< 0.0001
2020	325	272	215	201	206	196	173		
% change 2020 vs. 2019	6.2%	− 31.3%	− 45.4%	− 51.9%	− 45.6%	− 47.6%	− 53.1%		
Second wave vs. first wave									
First wave	206	335	204	79	66	64	47	174.6	< 0.0001
Second wave	325	272	215	201	206	196	173		
% change second wave vs. first wave	57.8%	− 18.8%	5.4%	154.4%	212.1%	206.2%	268.1%		

Comparison in the number of weekly admissions between the period October 26th–December 13th, 2020, and the same timeframe in 2019 (2020 vs. 2019) and between October 26th and December 13th, 2020, and February 24th and April 10th, 2020 (second wave vs. first wave). Absolute values and the respective % changes are reported. Data were analysed through the Pearson’s Chi-squared test. Chi-squared tests and *p* values are shown. *p* < 0.05 is considered statistically significant

**Table 3:** Admissions per type in 2020 vs.2019 and in second wave vs. first wave

	Type of admission	No. per type of admission	No. of admissions	% of admissions	*χ*^2^ test df (1)	*p* value
2020 vs.2019						
2019	Planned	2058	2211	93.1%	176.1	< 0.0001
	Urgent	153		6.9%		
2020	Planned	1193	1525	78.2%		
	Urgent	332		21.8%		
Second wave vs. first wave						
First wave	Planned	664	932	71.2%	15.3	< 0.0001
	Urgent	268		28.8%		
Second wave	Planned	1193	1525	78.2%		
	Urgent	332		21.8%		

Comparison in the number of planned and urgent surgeries between the period October 26th–December 13th, 2020, and same timeframe in 2019 (2020 vs. 2019) and between October 26th and December 13th, 2020, and February 24th and April 10th, 2020 (second wave vs. first wave). Data are reported as absolute and percentage values, and compared through the Pearson’s Chi-squared test. Respective Chi-squared test and *p* values are shown. *p* < 0.05 is considered statistically significant

**Fig. 3:**
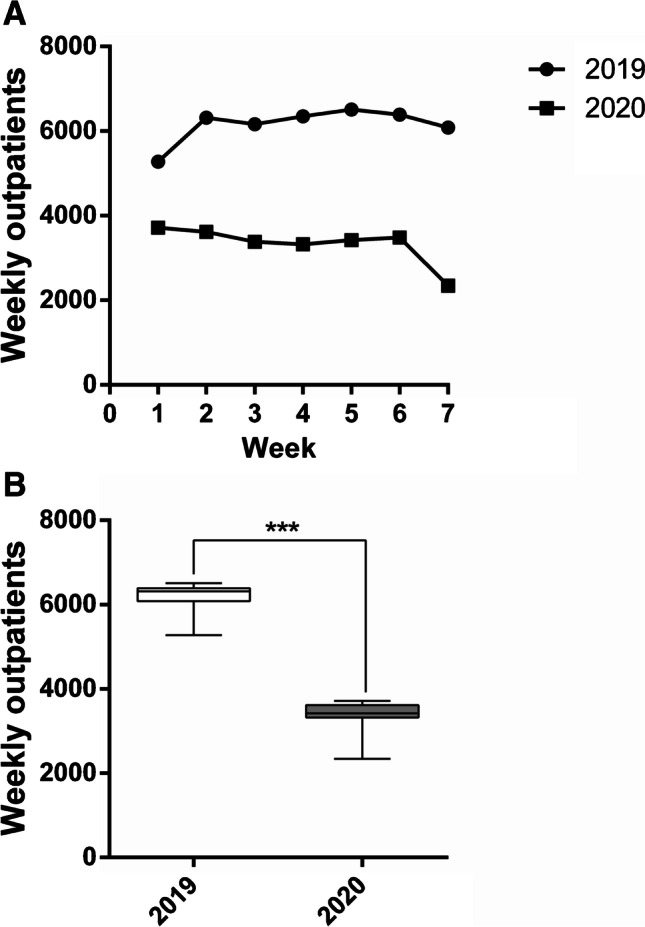
Outpatients from October 26th to December 13th, 2020, compared to the corresponding period in 2019. **A** Weekly outpatient trends during the considered 7-week timeframe in 2020 and 2019. **B** Outpatients weekly mean number in 2020 compared to 2019. Data are reported as minimum to maximum, and were compared trough the Mann–Whitney *U* test. Comparison is significant when *p* < 0.05

In the outpatients department, the total number of visits during the 7 weeks in 2020 (23,291) decreased by 45.9% compared to 2019 (43,092) (Table 4). Also, the weekly number of visits was significantly decreased from 6156.0 ± 412.5 patients, on average, in 2019, to 3327.3 ± 456.1 patients, on average, in 2020 (*p* = 0.0006), as shown in Figs. 3A and B.

**Table 4:** Weekly outpatients in 2020 vs.2019 and in second wave vs. first wave

	Week 1	Week 2	Week 3	Week 4	Week 5	Week 6	Week 7	Total
2020 vs. 2019								
2019	5278	6317	6161	6350	6513	6388	6085	43,092
2020	3715	3619	3386	3321	3426	3484	2340	23,291
% change 2020 vs. 2019	− 29.6%	− 42.7%	− 45.0%	− 47.7%	− 47.4%	− 45.5%	− 61.5%	− 45.9%
Second wave vs. first wave								
First wave	8670	5927	6352	2692	761	383	365	25,150
Second wave	3715	3619	3386	3321	3426	3484	2340	23,291
% change second wave vs. first wave	− 57.2%	− 38.9%	− 46.7%	23.4%	350.2%	809.7%	541.1%	− 7.4%

Comparison in the number of weekly outpatients between the period October 26th-December 13th, 2020, and the same timeframe in 2019 (2020 vs. 2019) and between October 26th and December 13th, 2020, and February 24th and April 10th 2020 (second wave vs. first wave). Absolute values and the respective % changes are reported

The number of patients admitted to triage procedures was reduced, by 37%, in 2020 compared to the corresponding period of 2019. However, in relative terms, while there was a consistent reduction in the proportion of subjects admitted with both white (− 41.6%) and green triage codes (− 52%), the milder reasons for admissions as described in previous papers [3–11], a net and consistent, increase in the number of yellow (from 57, in 2019, to 494, in 2020; + 766.7%) and red cases (from 1, in 2019, to 5, in 2020; + 400.0%), was recorded (Table 5).

**Table 5:** Admissions in emergency room in 2020 vs.2019 and in second wave vs. first wave

2020 vs. 2019					
Triage Code	2019	2020	%	Incidence on total cases	
				2019	2020
White	730	426	− 41.6%	20.0%	18.5%
Green	2864	1375	− 52.0%	78.4%	59.8%
Yellow	57	494	766.7%	1.6%	21.5%
Red	1	5	400.0%	0%	0%
Total	3652	2300	− 37.0%		
Second wave vs. first wave					
Triage Code	First wave	Second wave	%	Incidence on total cases	
				I wave	II wave
White	252	426	69.0%	18.9%	18.5%
Green	1016	1375	35.3%	76.1%	59.8%
Yellow	60	494	723.3%	4.5%	21.5%
Red	7	5	− 28.6%	0.%	0.%
Total	1335	2300	72.3%		

Comparison in the number of admissions in emergency room between October 26th–December 13th, 2020, and October 26th–December 13th, 2019 (2020 vs. 2019) and between October 26th–December 13th, 2020, and February 24th–April 10th, 2020 (second wave vs. first wave). Data are reported as absolute and percentage values (incidence on total cases); also, the respective % change in 2020 vs. 2019 and second wave vs. first wave is reported

### Comparison between first and second wave

The relative impact of the seven week period of each of the two pandemic waves (February 24th to April 10th, 2020 vs. October 26th to December 13th, 2020) was then evaluated.

**Fig. 4:**
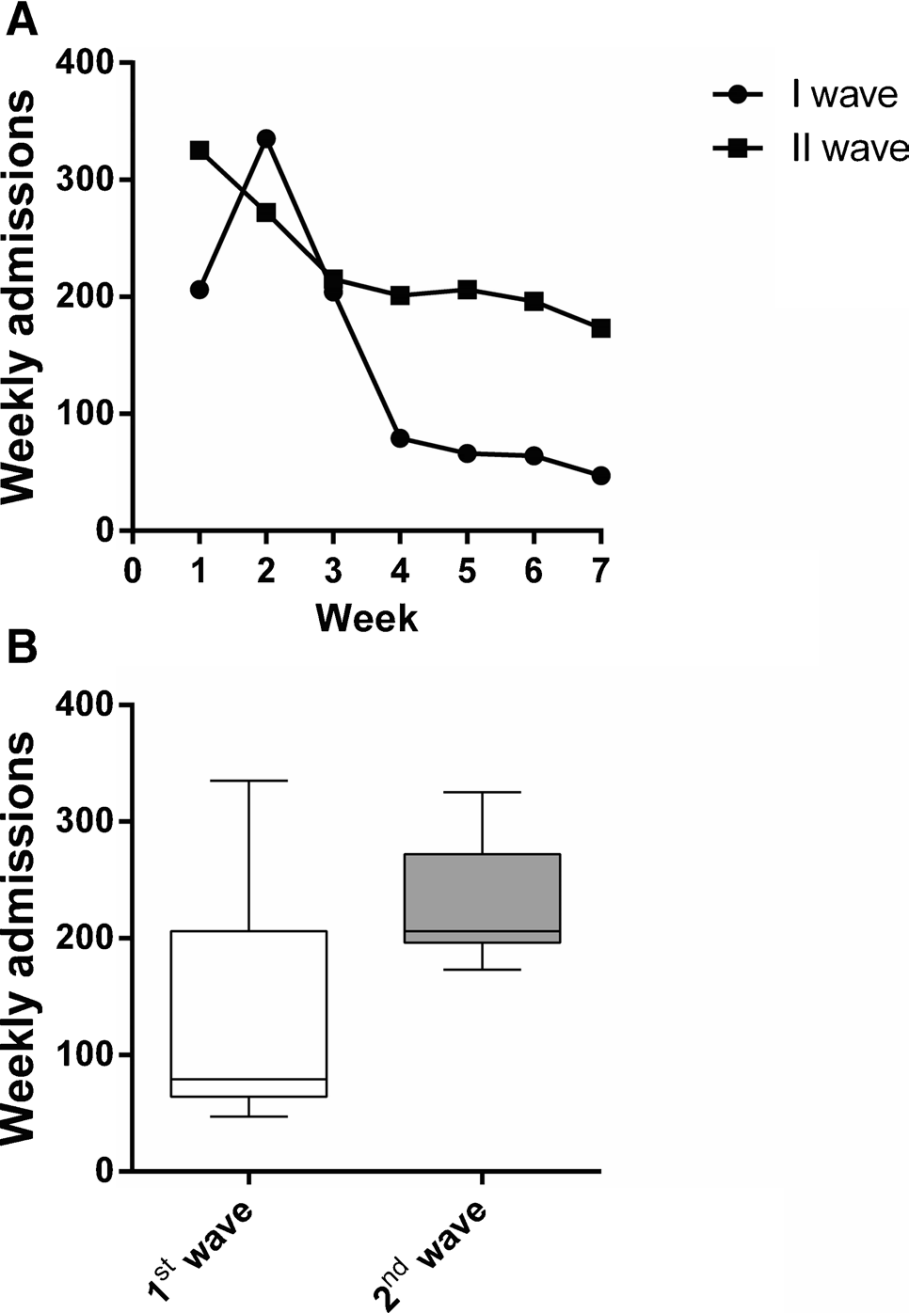
Admissions from February 24th to April 10th, 2020 (first wave) compared to admissions from October 26th to December 13th, 2020 (second wave). **A** Weekly admission trends during the first wave and the second wave. **B** Mean number of weekly admissions in the first wave compared to the second wave. Data are reported as minimum to maximum, and were compared trough the Mann–Whitney *U* test. Comparison is significant when *p* < 0.05

According to the data reported in Table 1, the total number of admissions during the second wave (1588) was significantly increased by 58.6% compared to the first wave (1001) (*p* = 0.001) (Table 1). By looking at the weekly trends, a greater drop was recorded during the first wave, especially due to the abrupt decrease between the third and the fourth week, compared to the second wave, when the decrement was, instead, smoother and more consistent in the first three weeks of the period (Table 2; Fig. 4A). However, although the mean weekly number of admissions during the two waves differed, the statistically significance was not reached (143.0 ± 108.0 in the first wave, 226.9 ± 52.9 in the second wave; *p* = 0.176; Fig. 4B).

The disproportion in favour of surgical admissions than rehabilitations, observed during the first wave, was further pushed in the second wave with an increase by 63.6% in the former and a decrease by 8.7% in the latter activities (Table 1). As shown in Table 3, the increase in surgical admissions during the second wave depended from both elective (+ 79.7%: 1193, in the second wave, vs. 664, in the first wave) and emergency surgeries (+ 23.9%: 332 vs. 268).

Considering all the emergency cases, the number of surgeries performed in SARS-CoV-2 patients was relatively small and comparable during both waves (11.6% during the first wave and 11.7% during the second wave) (Table 6). The percentage of SARS-CoV-2 + surgery remained roughly constant during the seven weeks of the second wave (Table 7).

**Table 6:** Number of surgeries performed in SARS-CoV-2 positive and SARS-CoV-2 negative patients in second wave vs. first wave

	Type of surgery	No. per type of surgery	No. of surgery	% of surgery	*χ*^2^ test df (1)	*p* value
First wave	SARS-CoV-2 +	31	268	11.6%	0.005	0.473
	SARS-CoV-2 −	237		88.4%		
Second wave	SARS-CoV-2 +	39	332	11.7%		
	SARS-CoV-2 −	293		88.3%		

Comparison in the number of surgeries performed in SARS-CoV-2 positive (SARS-CoV-2 +) and SARS-CoV-2 negative (SARS-CoV-2 −) patients between October 26th–December 13th, 2020, and February 24th–April 10th, 2020 (second wave vs. first wave). Data are reported as absolute and percentage values, and are compared through the Pearson’s Chi-squared test. Respective Chi-squared test and *p* values are shown. *p* < 0.05 is considered statistically significant

**Table 7:** Weekly number of surgery performed in SARS-CoV-2 positive and SARS-CoV-2 negative patients from October 26 to December 13, 2020

	Week 1	Week 2	Week 3	Week 4	Week 5	Week 6	Week 7	Total
Second wave								
SARS-CoV-2 +	4 (8.7%)	7 (11.9%)	5 (13.2%)	10 (16.1%)	5 (10.4%)	4 (12.5%)	4 (8.5%)	39
SARS-CoV-2-	42 (91.3%)	52 (88.1%)	33 (86.8%)	52 (83.9%)	43 (89.6%)	28 (87.5%)	43 (91.5%)	293

Comparison in the number of weekly surgeries in SARS-CoV-2 positive (SARS-CoV-2 +) and SARS-CoV-2 negative (SARS-CoV-2-) from October 26th to December 13th, 2020. Absolute values and the respective percentage are reported for every week

**Fig. 5:**
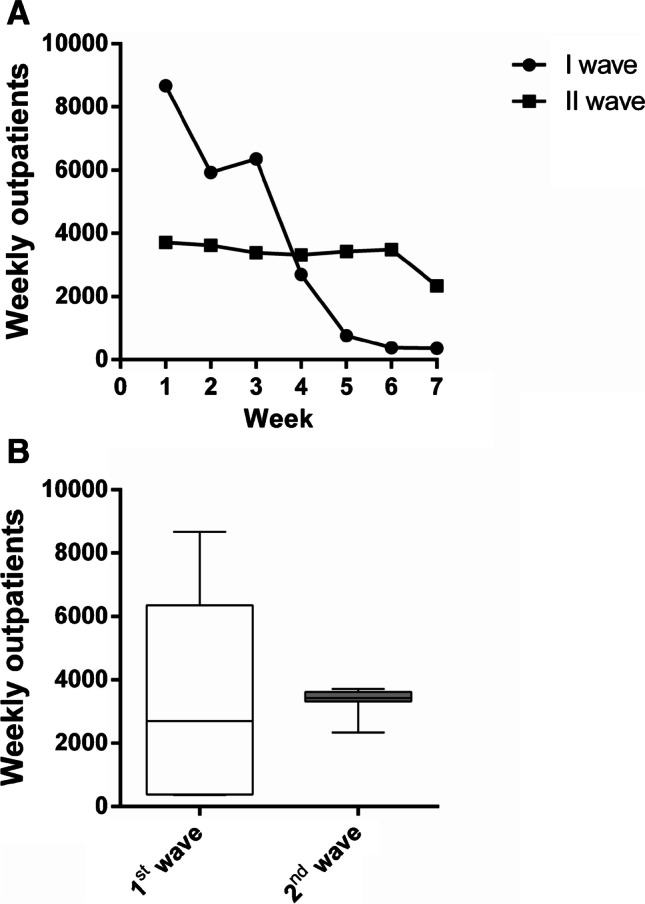
Outpatients from February 24th to April 10th, 2020 (first wave) compared to outpatients from October 26th to December 13th, 2020 (second wave). **A** Weekly outpatient trends during the first wave and the second wave. **B** Mean number of weekly outpatients in the first wave compared to the second wave. Data are reported as minimum to maximum, and were compared trough the Mann–Whitney *U* test. Comparison is significant when *p* < 0.05

Compared to the relative constancy of the weekly number of outpatients experienced in the second wave, with just a more consistent decrease between the sixth and the seventh week, during the first wave, the decline was definitively and important (Table 4; Fig. 5A), although the mean weekly volumes of the two waves were not statistically different (3592.9 ± 3376.2 in the first wave, 3327.3 ± 456.1in the second wave; *p* = 0.779; Fig. 5B).

Patient admission to ER triage procedure was increased during the second wave compared to the first wave (+ 72.3%) (*p* = 0.001). This increase was recorded for all the triage codes, but the red one (Table 5).

## Discussion

The first wave of SARS-CoV-2 infection hit Europe between March and May, 2020, and it was followed by a reduction of new cases during late spring–summer i.e. June to September. A second epidemic wave was foreseen [10] and finally took place starting in October–November and expanding into the beginning of 2021. The purpose of this study was to analyse the impact of a second pandemic wave in an orthopaedic referral centre during a seven week period of the second wave to be contextualized within the pandemic (i.e. comparison with the first wave) and compared with the previous non-pandemic period (i.e. comparison with the corresponding period in 2019).

The experience gained during the first wave [3] allowed a rapid redeployment of the logistics and personnel as soon as the regional health authorities increased the level of the organisational health structure to cope with the rapid increment of COVID cases. A dedicated ward of the hospital was re-expanded to receive patients from the ER department, adapted as a filtering area, and to accept SARS-CoV-2 positive patients already diagnosed; however, this screening and filtering activities never stopped since the first wave. A section of the operating block was still devoted at accepting SARS-CoV-2 positive surgery [5] and, after a period of substantial inactivity that followed the first wave and saw an operating room (OR) kept in stand-by, it started again to host SARS-CoV-2 positive cases regularly admitted from the ER, while a separate block continued to run both elective and emergency surgery in not infected patients. The intensive care unit (ICU) was preserved for SARS-CoV-2 negative patients, differently from the first wave when it was implemented and converted to treat SARS-CoV-2 positive patients requiring respiratory assistance, during the period of highest stress for the whole regional health system.

Unlike the first wave period, certain volumes of elective surgery were allowed, at this time. There was a smoother decline along the seven weeks during the second wave than during the first wave when, instead, this activity was completely stopped in the last three weeks, but the treatment of malignancies. Anyway, the volume of planned surgery dropped down by 42.0%, compared to the same period in 2019. This reduction was mainly due to the reduced number of slots made available to each surgical crew. The lack of staff tested positive and in quarantine, further decreased the availability of ORs and beds thus causing a consistent reduction of the activity. Moreover, orthopaedics patients experiencing minor symptoms spontaneously postponed surgery to safer times being afraid of COVID-19 or due to the impossibility of being assisted by relatives during their journey, as the relatives were not admitted in the wards based on safety rules. The most severe and painful cases were mainly treated: septic arthritis, bone necrosis, total joint arthroplasty (TJA) dislocation and loosing were predominant.

Emergency cases, mainly femoral neck fractures, increased due to an important reduction of OR availability in nearby hospitals. They were more than doubled compared to 2019 (332 vs. 153) when they counted for 6.9% of admissions while during the second wave they accounted for 21.8% of the surgeries, even exceeding those of the first wave (268). Great efforts were spent to maintain the surgical procedure within 48 hours from admission. ER department had to sustain the remarkable increased number of yellow priority codes, mainly related to trauma referred from other hospitals, much higher than during normal times, but also higher than during the first wave. While white and green priority codes were reduced by a half compared to 2019, they were increased compared to the first wave. Maybe, subjects avoided to present to ER in case of mild symptoms being afraid of the risks of infection. Moreover, social restrictions and confinement, that are currently ongoing in the country, have limited several activities, such as sports and recreational, that can expose people to minor traumatic events, that are typical reasons for ER admissions in an orthopaedic hospital.

The rehabilitation department, whose activity is strictly connected to those of the surgical department, for post-acute rehabilitation, continued to experience a strong reduction of bed availability and, hence, of inpatient admission. The dramatic decline recorded during the first wave (69 patients, 6.9% of all admissions) went further reducing the rate of admissions for rehabilitation to 4% (63 patients) during the second wave (in 2019, they were 425 corresponding to 16.1% of total admissions). Patients had either to be transferred to external rehabilitation structures or kept in surgical ward for longer and, at least, until the achievement of an acceptable degree of autonomy before being discharged at home. Meanwhile, home-based assistance facilities were implemented by local health authorities. Moreover, patients, and their relatives, showed an increasing willingness to be discharged at home as soon as possible in order to limit the nosocomial permanence and, consequently, the supposed and feared risks of infection.

All patients underwent to nasopharyngeal swab PCR test to check for SARS-CoV-2 infection before admittance and elective surgery was suspended in case of positive test. SARS-CoV-2 positive subjects were treated only in case of emergency and the surgery was performed in a dedicated OR with adequate safety devices. The overall number of SARS-CoV-2 positive patients that were treated during the second wave was greater than during the first wave (39 vs. 31) although the relative numbers were very similar (11.7% vs. 11.6% of the emergencies). It is possible that during the first wave, the number of SARS-CoV-2 subjects was underestimated as the test was performed in all the patients since week five, while in the first weeks, it was done only in symptomatic patients. Anyway, the number of positive patients surgically treated was a minority.

According to the local and regional requests aimed at reorganizing the needs of the networking care and based on the numbers here presented, it appears clear that most of the activity has been devoted to the treatment of the urgent orthopaedic trauma activity. Elective surgery specialists reduced their activity but, differently from the first wave, they did not stop it and they were not moved to the traumatology activity.

The outpatients department suffered a 46% reduction in the number of consultations when compared to the activity in 2019. However, this reduction was limited during the second wave compared to the first wave, when it was nearly abolished. Nevertheless, the extension of the waiting lists is still experienced although there is a high degree of drop-out of patients at the planned follow-up post-surgery visits. As a consequence, the nearby future sees a real risk of neglected or delayed diagnosis, for instance, implant loosening, periprosthetic occult infection or wear, that could lead to the increase in the number of complicated cases to be addressed.

COVID-19 epidemic is still heavily affecting the health system and the hospital structures while medical and surgical activities continue to be severely disrupted as shown by this observational analysis of a seven week period during the second wave. Orthopaedic departments are still under pressure and the return to pre-pandemic volumes is still unforeseeable. The reduction in the number of elective surgery is determining a steep extension of the waiting lists and, in turns, it increases the anxiety in patients in need of surgical procedures, like spine and prosthetic surgery. These procedures, indeed, have been defined as not urgent, although they are addressed to the treatment of patients experiencing limitation in autonomy and chronic, and sometimes severe, pain leading to drugs overuse.

Psychological concerns in healthcare personnel are to be considered, too. Repeated reorganizations of wards and duties undermine mood and motivation, especially in front-line health workers and have important repercussions on working capacity. Moreover, the forced disruption of the teams leads to loss of efficiency, time extension of work paths, and increased risk of mistakes out of protocols.

On the other side, the real economic impact of the pandemic on single health-related activities is still to be fully assessed. Elective procedures, for instance, account for the majority of the hospital costs but also revenues. The steep reduction of total admissions, with a more or less unique focus on urgent surgeries and complex cases, is likely to lead to the generation of an important economic burden in hospital budgets. Moreover, the costs related to the frequent reorganization of hospital wards and activities, protective and safety devices and protocol implementation are supposed to increase.

Although aimed at merely describe a contingent situation, this study is not exempt from limitations. First of all, it is a retrospective evaluation, but there was no other way to compare data in a prospective way due to the unexpected nature of the events. Secondly, it describes the unique condition of a referral orthopaedic centre, which can differ from general hospitals, as well as from other specialized centres. To the best of our knowledge, this is the first paper that describes, in details, how the activities changed in such a specialized hospital during the second wave of SARS-CoV-2 outbreak by considering such changes during the first wave and by comparing the situation with that of normal period (i.e. 2019). Finally, we analysed also the administrative data flow, but there was no other chance to collect such a great number of cases from the entire institution. The database was double checked with clinical data flow and compared with the corresponding one of 2019 and of the seven week period during the first wave.

In conclusion, the analysis of a seven week period during the second pandemic wave in an orthopaedic centre in Northern Italy brought into light the issues that have been already experienced during the first wave and the perception that a long period is still ahead before normal activities can be resumed. The increase of urgent orthopaedic and trauma activities coupled with the reduced possibility to deliver elective surgery and the reduced volume of outpatients was the more critical aspects that have emerged even during the second wave. The number of treated SARS-CoV-2 positive patients did not significantly increase compared to the first wave even in the presence of a sound screening. Meanwhile, the extension of the waiting lists and the increase in the number of neglected cases with more complicated settings are expected in the next future. Mass vaccination and social confinement are, at the present, the strongest weapons to face up the nearby future.

## Data Availability

No repositories were used. Data and material are available at the corresponding author.
